# A perspective on proteomics in cell biology

**DOI:** 10.1016/j.tcb.2013.10.010

**Published:** 2014-04

**Authors:** Yasmeen Ahmad, Angus I. Lamond

**Affiliations:** Centre for Gene Regulation and Expression, College of Life Sciences, University of Dundee, Dow Street, Dundee DD1 5EH, Scotland, UK

## Abstract

During the past 15 years mass spectrometry (MS)-based analyses have become established as the method of choice for direct protein identification and measurement. Owing to the remarkable improvements in the sensitivity and resolution of MS instruments, this technology has revolutionised the opportunities available for the system-wide characterisation of proteins, with wide applications across virtually the whole of cell biology. In this article we provide a perspective on the current state of the art and discuss how the future of cell biology research may benefit from further developments and applications in the field of MS and proteomics, highlighting the major challenges ahead for the community in organising the effective sharing and integration of the resulting data mountain.

## Highlights

• Proteomic strategies facilitate system-wide analyses of protein complexes.
• Isotope labelling allows quantitative measurement of protein properties, not only their identification.
• There is a major need to organise effective community sharing of the proteomic data mountain.
• The integration of proteomic data with other online data repositories must be improved.

## Introduction

In 1997 a commentary in *Trends in Cell Biology*, entitled ‘Cell Biology and the genome projects – a concerted strategy for characterizing multiprotein complexes by using mass spectrometry’ [1], reviewed some of the exciting technical developments that had allowed the use of MS to identify proteins, particularly when used in concert with the growing collection of DNA sequence information. At that time this largely comprised libraries of expressed sequence tag (EST) clones. It highlighted the promise this held for cell biology, showing how MS could greatly enhance the efficiency and sensitivity of protein detection over previous methods, and hence facilitate the direct analysis of proteins and multiprotein complexes involved in biological responses and regulatory mechanisms. This article, which pre-dated completion of the human genome project, also anticipated that MS-based proteomics would grow to provide the method of choice for protein analysis and for deciphering the functions of open reading frames (ORFs) as more genome sequences became available.

Fifteen years later, the efficient detection of cell proteins using MS has indeed become routine, and it is hard to imagine conducting biological research without access to complete genome sequences. The speed and resolution of mass spectrometers has increased dramatically, and we now have access to powerful software for automated analysis of raw spectra 2, 3, 4, 5. In this article we discuss how modern MS-based proteomics can be used to study many areas of cell biology. We also look ahead to the next 15 years, illustrating new opportunities for advancing cell and molecular biology using MS-based proteomic strategies. One of the key future challenges we foresee is the need for the cell biology community to develop a coherent strategy, as well as new computational tools, to cope with the effective integration, analysis, and sharing of the emerging proteomics ‘data mountain’.

## From genomes to multi-dimensional proteomes

Now that the human genome is sequenced, together with the genomes of most common model organisms, it is tempting to assume that cell ‘proteomes’ – in other words a detailed inventory of the proteins present – can be deduced simply by reference to ORFs in the corresponding DNA sequence. In practice the situation is much more complex. For example, in higher organisms there is usually no simple ‘one-to-one’ relationship between genes and proteins. Instead, there are ‘one-to-many’ relationships, primarily because a single ORF can encode multiple protein isoforms.

A range of mechanisms, including alternative splicing of mRNA precursors, cleavage and processing of polypeptide chains, and post-translational modifications (PTMs), contribute to generating multiple protein isoforms, with distinct or overlapping functions. To complicate the situation further, the same polypeptide chains can also form distinct functional pools of protein that are regulated independently. For example, the catalytic subunit of protein phosphatase 1 (PP1) forms many separate protein phosphatase enzymes by binding to an array of different targeting subunits. These distinct and independently regulated forms of protein phosphatases act on different substrates in different subcellular locations [6].

**Figure 1:**
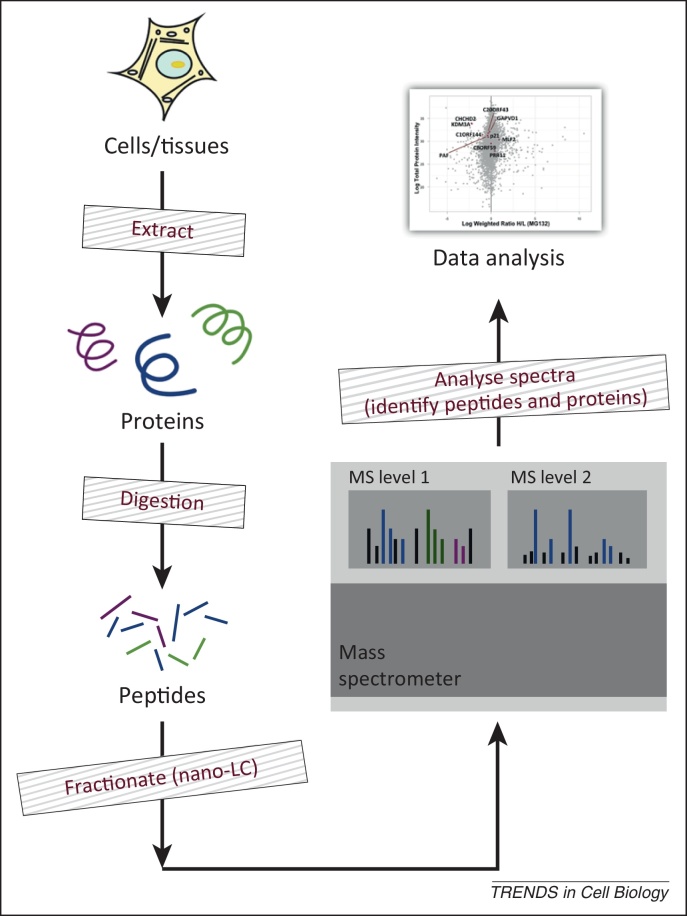
Bottom-up proteomics workflow. ‘Proteomics’ is used throughout as an umbrella term for the large-scale identification and analysis of proteins. We focus here specifically on the analysis of proteins by mass spectrometry (MS), because this has emerged as by far the most widely used and efficient current technology. The standard ‘bottom-up’ proteomics workflow illustrated involves isolating proteins from either cells or tissues, digesting them to peptides using one or more proteases (e.g., trypsin), then separating the resulting peptide mixtures by nano-LC (nano-liquid chromatography) and identifying the peptides in a mass spectrometer. The resulting peptide identifications are subsequently mapped to proteins, using genomic information to identify open reading frames (ORFs) that encode these peptides. Although the MS analysis actually measures peptides, most subsequent data analysis in cell biology experiments interprets the results in terms of the inferred protein identifications, and the quality of the data can vary according to how many peptides were detected for each protein. Usually a minimum of at least two separate peptides are required to confirm protein identification.

Thus, although genomic sequences reveal the protein-coding potential for a given organism or species, they do not reliably inform us about the many protein properties that correspond to the variables that are usually modulated during biological responses and regulatory mechanisms. The dynamic nature of these protein properties also means that they cannot be deduced from a static DNA genome sequence alone, either for a given cell cycle stage or at different times during response to a cell signalling event. Furthermore, in most cases measurements of transcript levels will not reveal dynamic protein properties, such as PTM patterns, interaction partners, or subcellular localisation, let alone the existence of distinct functional pools of protein. Indeed, recent studies indicate that transcript levels often do not reliably reflect the abundance of the cognate protein [7]. Thus, crucial information on protein expression and interactions, which is central to a detailed understanding of cell phenotypes and molecular mechanisms, will not emerge from analysis of genomic and transcriptomic datasets alone. Therefore, to evaluate how protein properties vary over time during cellular responses one must directly and empirically measure and quantitate proteins at multiple time-points. The method of choice for such direct protein measurement is now ‘bottom-up’ MS-based proteomics (Figure 1).

## Time-lapse proteomics – quantitating proteome responses

It is clear that proteomes change significantly in real time, making them highly dynamic. For example, the changing nature of an organelle proteome was demonstrated by comparing the protein composition of nucleoli isolated from cells following drug treatment to inhibit transcription 8, 9. This required multiple MS analyses, using nucleoli purified from cells isolated under different conditions and at different time-points, thereby creating a ‘time-lapse’ proteomics view of the response. The synaptosomal proteome provides another example because it was shown to change during postnatal development using the multi-dimensional protein identification technology (MudPIT) [10]. With improvements in the speed and resolution of MS workflows it is now possible to perform such time-lapse proteomics studies to capture not only protein identities but also a wide range of protein properties for many thousands of cell proteins at multiple time-points. Expanding such studies on a system-wide scale, encompassing measurement of a large fraction of the expressed cell proteome, promises to provide unprecedented new insights into many of the molecular mechanisms involved in cellular regulation. However, collecting multi-dimensional data at multiple time-points results in an explosion in the volume of proteomics data, and this creates challenges in effective data management.

**Figure 2:**
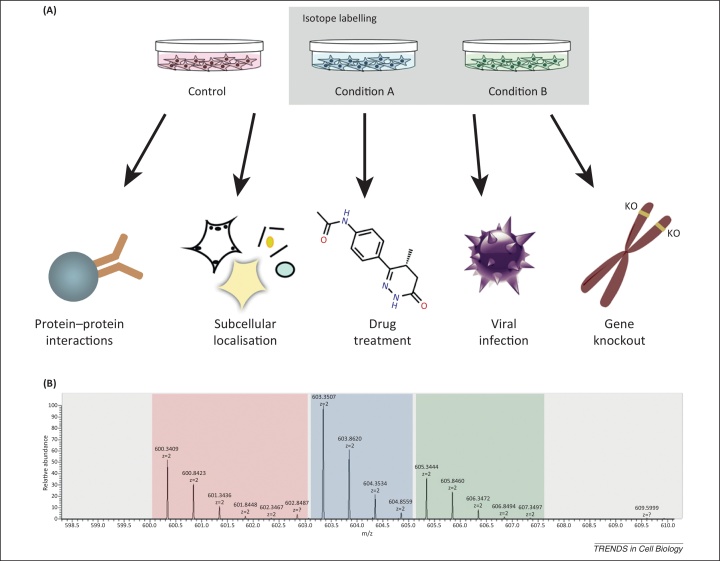
Isotope labelling strategies. Isotope labelling methods such as SILAC (stable isotope labelling with amino acids in cell culture) and iTRAQ (isobaric tag for relative and absolute quantitation) provide a convenient approach for the quantitative proteomic comparison of two or more experimental variables by introducing tags that can be discriminated in the mass spectrometer to distinguish and measure the proteins from each separate condition or cell sample. **(A)** Isotope labelling is a highly flexible strategy that can be adapted to identify and compare protein interaction partners, subcellular protein localisation, drug treatment, viral infection, and effects of genotype etc. **(B)** A multispectral image (MSI) spectrum of a peptide selected in a triple SILAC experiment. In the illustrated example the *m*/*z* signal from the peptide (*x* axis) is separated in the spectrum into three clusters of signals, corresponding to the ‘light’, ‘medium’, and ‘heavy’ isotopic forms. The measured ion intensities (*y* axis) for each isotopic form of the peptide are then compared, and these reflect the corresponding property of the protein from which the peptide was derived in each cell state. In most cases, data are subsequently represented by averaging the separate values measured for all peptides identified for each protein.

One of the most successful MS-based strategies for time-lapse proteomics has been development of isotope-labelling methods that facilitate the quantitation and differential comparison of proteins, either at different times and/or under different conditions. This is a flexible approach for comparing a control sample with one or more varied experimental conditions, such as changes in protein levels and PTMs in response to drug treatment, viral infection, hormone stimulation, cell differentiation, or oncogene activation (Figure 2A). It has also been adapted to provide a quantitative assay for evaluation of protein properties, including subcellular protein localisation, rates of protein synthesis and degradation, and for discrimination of specific from non-specific protein interaction partners 11, 12, 13, 14.

## Isotope-labelling strategies

Some of the first examples of using metabolic labelling to incorporate stable isotopes into proteins for quantitative proteomics took advantage of labelling methods previously used by structural biologists for nuclear magnetic resonance (NMR) analyses 15, 16. Subsequently, the differential isotope labelling strategy termed SILAC (stable isotope labelling with amino acids in cell culture) [17], which involves metabolic labelling of proteins *in vivo*, has been widely applied to cell biology. Thus, translation of proteins incorporates amino acids that have the natural ‘light’ isotopes of carbon, nitrogen, or hydrogen substituted with heavier isotopic forms such as ^13^C, ^15^N, and/or ^2^H. Usually this is carried out using isotope-substituted forms of arginine and/or lysine because subsequent trypsin digestion of the isolated proteins for MS analysis, which cleaves at basic residues, generates peptides with a single labelled amino acid, thereby simplifying analysis and quantification. The distinct ‘light’, ‘medium’, and ‘heavy’ forms of each peptide detected by MS reflect the relative amounts of the corresponding protein in each of the three ‘isotopically encoded’ cell populations.

The light, medium, and heavy forms of the same peptides can be resolved and quantitated simultaneously within the same sample by MS, thereby reducing potential sources of experimental variation between samples. A similar strategy has also been applied to a range of model organisms including yeast, bacteria, nematodes, plants, and mice 10, 18, 19, 20. We anticipate that further studies using whole-organism metabolic labelling will provide important new opportunities for linking quantitative proteomics data with physiology and genetic approaches.

Although the utility of the SILAC proteomics approach has already had a major impact in cell biology, it is worthwhile to consider its potential limitations. For example, although metabolic labelling is an effective way to uniformly incorporate isotopic tags into proteins, in practice it is not always feasible, as seen with clinical samples and some model organisms. Also, the ion intensity for each peptide quantitated by duplex or triplex SILAC is distributed between several, separate isotopic peaks in the spectrum (Figure 2B). This can lower the total number of peptide identifications from a given sample owing to the complexity introduced by the multiple isotopic peaks in the MS level 1 spectra. This complexity tends to limit the scope for extensive multiplexing beyond two or three different conditions in parallel.

An alternative approach is to use a chemical, rather than metabolic, labelling strategy, which can be applied to any isolated protein samples, including human clinical material. Samples are quantified separately, as seen with a technique such as ICAT (isotope-coded affinity tags) [21] which attaches an isotope-coded tag to the sulfhydryl groups of cysteine residues on peptides. This has the limitation, however, of only labelling peptides that include cysteine residues. By contrast, dimethyl labelling, which attaches isotope-substituted formaldehyde to label the amino termini of peptides and the ɛ-amino groups of lysines, provides a more efficient and cost-effective strategy for chemical tagging of peptides that may come to be more widely used in future. One disadvantage of this method is that lysine tagging may mask PTMs that specifically occur on lysine amino acids.

Another variation on the chemical labelling strategy uses isobaric tags to label isolated peptides, as seen in techniques such as iTRAQ (isobaric tag for relative and absolute quantitation) [22] and TMT (tandem mass tags) 23, 24. Because the tags employed are isobaric, multiple different labelled samples can be combined for MS analysis, and the separate labelled forms are resolved and quantitated based on the different reporter ions they generate after the peptides are fragmented by collisional dissociation in the mass spectrometer. This has the advantage of allowing a higher degree of multiplexing, with eight or more different combinations. Hence, complex experiments can be performed and analysed in parallel. For example, changes in protein levels can be compared at multiple time-points for both a control and drug-treated sample. In practice, these isobaric tagging methods have proven difficult to apply for the accurate quantitation of proteins in complex biological samples, although recent improvements in MS instrumentation have helped to improve this situation.

In comparison with metabolic labelling, all of the methods described above that are based upon chemical attachment of tags to peptides introduce additional steps in the analytical strategy that are potential sources of variation and experimental error. By contrast, metabolic labelling methods allow separate cell samples to be mixed at an early stage and proteins isolated simultaneously, and this helps to improve the accuracy of analysis and quantitation. In this regard it is interesting that a new method, known as NeuCode SILAC [25], appears to offer a ‘best of both worlds’ combination of the SILAC metabolic labelling strategy with the high multiplexing opportunities of using multiple isobaric tags. The NeuCode SILAC method exploits the remarkable efficiency and resolution of the latest generation of MS instruments to distinguish between isotopes of similar mass, based upon differences in nuclear binding energy. Using this approach it has been possible to grow cells with different forms of lysine substituted with ‘isotopomers’, each differing in effective mass in the tens of milliDalton range, and to distinguish and quantitate peptides derived from these differentially labelled proteins.

It is reported that multiplexing by NeuCode SILAC can detect as many as 12 different isotopomer labels in a sample, and this could in principle be extended in future to detection of 20 or more multiplexed samples [25]. The signal dilution effect encountered in regular SILAC is avoided in this approach, allowing more efficient detection and quantitation of peptide ions in the mass spectrometer. At present, however, the challenge in applying NeuCode SILAC to cell biology is that it requires pushing the resolution of current MS instruments to their absolute limits. It also requires the requisite isotopomer-substituted amino acids and analytical software to become widely available to the cell biology community. However, given the exciting prospect this new technology offers for high multiplexing and for accurate and efficient quantitation of protein levels in many types of complex experiments, we anticipate that it will become widely adopted in the future. This will be aided especially when the next generations of MS instruments arrive that can routinely achieve the high mass-resolution needed.

## Analysis of protein complexes

**Figure 3:**
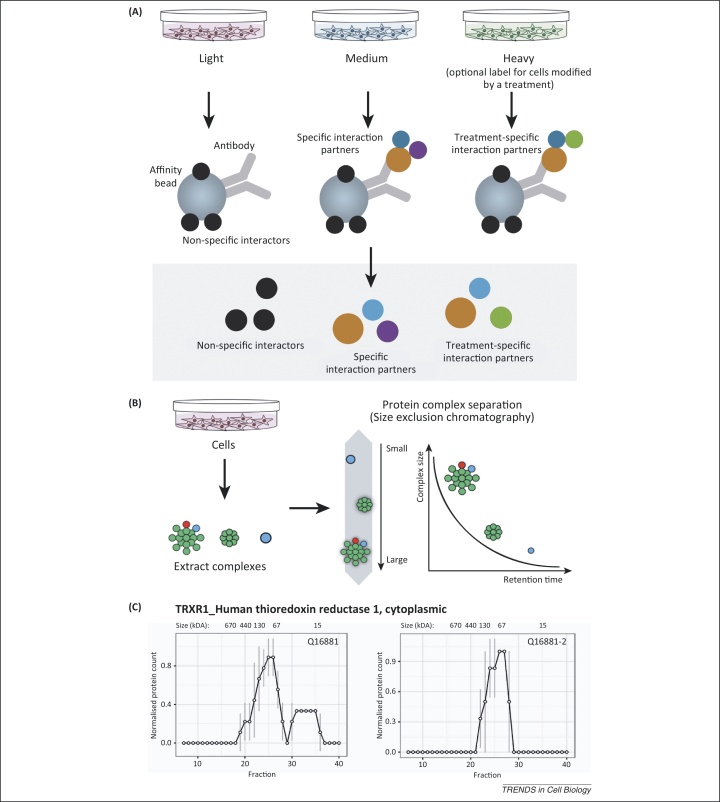
Approaches to protein interaction analysis. **(A)** Isotope labelling (e.g., SILAC) has been widely used as a method to discriminate reliably between specific and non-specific protein interaction partners in immunoprecipitation and affinity pull-down experiments. By measuring the ratio of light (control – e.g., non-specific Ig or no bait protein) to heavy (e.g., specific Ab or tagged bait protein) isotope-labelled proteins that are copurified and detected by MS analysis, proteins that bind non-specifically will typically have 1:1 H/L ratios whereas specific interaction partners will have high H/L ratios. Using a triple-labelling strategy this can be extended, for example, by using the comparison of M/L and H/L isotope ratios to compare specific binding either to different protein isoforms or mutants, or to compare binding in the presence or absence of an inhibitor, etc. **(B)** A high-throughput proteomics strategy for analysing protein complexes by (i) first separating protein complexes present in cell extracts using a chromatography method such as SEC, then (ii) detecting essentially all of the proteins in each resultant SEC fraction following protease digestion and mass spectrometry. This highlights candidate protein components of complexes based upon coelution profiles across the SEC fractions, and can potentially distinguish complexes containing either specific protein isoforms and/or PTMs. It can be applied to the system-wide comparison of differences in cellular protein complexes under different growth conditions, or following drug treatments or other perturbations. **(C)** An example of a protein, TRXR1_HUMAN, which has multiple isoforms showing differing profiles of interaction. Abbreviations: Ab, antibody; H/L, heavy/light; Ig, immunoglobulin; M/L, medium/light; PTMs, post-translational modifications; SEC, size exclusion chromatography; SILAC, stable isotope labelling with amino acids in cell culture.

Most cellular proteins function as components of protein complexes rather than as single polypeptides. Therefore, characterisation of the composition and dynamics of multiprotein complexes is crucial in most areas of cell biology. A variety of methods and approaches are used to identify protein interaction partners, typically involving variations on immunoprecipitation (IP) and affinity pull-down procedures (Figure 3A), which can be combined with MudPIT 26, 27 to provide a gel-free analytical workflow. Not only has MS analysis provided a more sensitive way of detecting coprecipitated proteins in IP experiments, isotopic labelling strategies have also provided a powerful and unbiased methodology for reliably discriminating specific from non-specific interaction partners in pull-down experiments [28], as well as providing a convenient way to distinguish further details of protein complexes. For example, it can be used to determine isoform-specific protein interaction partners and to characterise differences in protein complexes, either under specific conditions, in different subcellular compartments, or after drug treatments or viral infection.

We expect that the role of MS-based IP experiments using both label-free and isotopic labelling strategies will continue to expand in the future. There is also considerable scope to improve the methodology further, for example by incorporating additional information when evaluating whether a coprecipitated protein is likely to be a specific interaction partner or not. Nonetheless, affinity pull-down of interacting proteins also has inherent limitations – including limited ability to resolve multiple related forms of a complex that share some components and are all isolated together by IP, but which differ in the presence of specific subunits and/or different protein isoforms or PTMs. Affinity purification is also difficult and costly to adapt for the system-wide analysis of large numbers of complexes and for the study of changes in protein complexes during different cellular growth conditions and responses. For system-wide studies, new approaches that combine large-scale separation of protein complexes in cell extracts, using either size exclusion chromatography (SEC) 29, 30 or other variants of high performance liquid chromatography (HPLC) [31], combined with efficient MS-based protein identification, offer promising ways to characterise protein complexes and their dynamics (Figure 3B). For example, recent studies have used SEC combined with MS to study changes in protein complexes in HeLa cytoplasmic extracts following epidermal growth factor stimulation [32], and have shown that specific forms of native complex can be resolved from U2OS cell extracts that are differentially associated with distinct protein isoforms and post-translationally modified proteins [33] (Figure 3C). We expect that further developments of these combined HPLC-based complex fractionation and MS-proteomics approaches will find widespread applications throughout cell biology.

## Proteomics ‘big data’ and super-experiments

There is likely to be continued growth in proteomics publications as the methods and scope for using MS-based approaches to address problems in cell biology develop further. We also anticipate the expansion of quantitative, ‘next-generation’ proteomics strategies for the systematic, quantitative analysis of protein properties – in other words the determination of ‘multi-dimensional proteomes’. The corollary is that the future value of proteomics in cell biology is closely linked with the need to develop effective, new strategies for multi-dimensional data analytics and to cope with the large volumes of proteomics data that will be generated. This includes the need to build new and more efficient data-processing pipelines which will be increasingly important to cope with the scale and complexity of future proteomics data arising from large-scale experiments.

**Figure 4:**
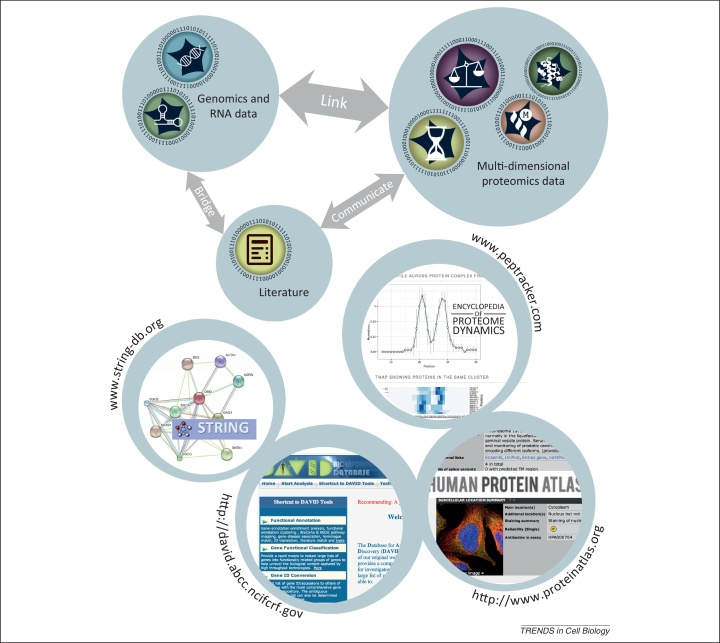
Data integration and resources for online sharing. The figure illustrates the need to link highly annotated, multi-dimensional proteomics data with information from large-scale, genomic and transcriptomic sequences and associated literature. Four publicly available online tools for sharing data related to proteomics are illustrated – STRING (Search Tool for the Retrieval of Interacting Genes), DAVID (Database for Annotation, Visualisation and Integrated Discovery), The Human Protein Atlas, and the Encyclopedia of Proteome Dynamics.

This brings forth several important challenges for the international research community if we are to exploit fully the opportunities that proteomics offers cell biology. We highlight in particular the need to tackle both effective data sharing, ideally through the creation of freely accessible, user-friendly, online data repositories and the related need to integrate proteomics data efficiently with other published information and with cognate large-scale genomic and transcriptomic data (Figure 4). The utility of integrated data analysis tools to evaluate and analyse new results is already seen with online resources such as PHOSIDA (Phosphorylation Site Database), a PTM database [34], STRING (Search Tool for the Retrieval of Interacting Genes) [35], DAVID (Database for Annotation, Visualisation and Integrated Discovery) [36], and The Human Protein Atlas [37].

There is currently a lack of any coordinated, international ‘open data’ strategy in the proteomics community and this is a situation that we hope will be addressed as a matter of urgency. Although public repositories are being developed for depositing raw MS data files, such as the PRIDE (Proteomics Identifications) database [38] and more recently CHORUS (Collaborations in HIV Outcomes Research – US; http://chorusproject.org), we suggest that this alone will not meet the needs of the cell biology community. In addition to raw file repositories, cell biologists require convenient access to searchable databases providing complex, multi-dimensional proteomics data at the level of identified protein sequences, including the measured values of protein properties in different cell types and under different conditions. The value of such data will be further increased if entries are extensively tagged with consistent metadata that can be filtered and selected.

We have initiated such a scheme by creating a proteomics open data resource, termed the ‘Encyclopedia of Proteome Dynamics’ (EPD) [13]. The EPD is a searchable database that provides access to large-scale proteomics datasets describing subcellular protein localisation, protein turnover rates, protein complexes, isoform expression patterns, and PTMs derived from studies on several human cell lines (http://peptracker.com/epd/). Expanding the EPD and other similar resources to integrate data from many different laboratories will require community-led initiatives to agree on common metadata standards. It will also require commitment from funding agencies to provide the resources needed to create and maintain such tools for effective data integration and sharing.

An advantage to sharing large datasets across the community in a searchable format, including detailed and consistent metadata annotations, is the opportunity to build collections of multi-dimensional data from many independent experiments. This can be organised to facilitate analysis of the whole dataset and cross-correlation between parameters and measurements recorded in many separate experiments. However, to integrate and mine datasets effectively it is imperative that the community agrees on definitions of metadata terms and how they are applied, using these terms consistently in publications and data collections. This would make it much easier and more accurate to combine large data collections. In this way the value of the combined data could be greater than the sum of the conclusions derived from the analysis of any individual set of experiments – because it allows conclusions to be reached through the comparison of different result sets. Thus, the combined data constitute a ‘super-experiment’ that can answer questions that potentially were not conceived during the generation of the individual experimental datasets contributing to it. For example, it can detect a difference in protein expression levels, or any other properties, which consistently correlate with genotypes across data from multiple different cell lines.

Multi-dimensional proteomics databases can be used to generate new hypotheses for future evaluation and to test the feasibility of new ideas against a large collection of available data. This may already either confirm, or disprove, a new hypothesis or research question without the need to perform additional experiments, thereby saving time and research funds. For example, if a model requires that two or more proteins interact to mediate a particular response or mechanism, this can be evaluated initially by checking whether these proteins are detected in the same subcellular compartments and whether they copurify and/or cofractionate by SEC analysis. These are data already available, at least for some human cell lines, in the EPD. Another example of a ‘super-experiment’ is the Protein Frequency Library (PFL) (http://peptracker.com/pfl/), which records data annotating all proteins from many coimmunoprecipitation experiments [12]. The PFL compares similar datasets, based on their metadata and the frequency of protein detection, to indicate whether proteins are specific or non-specific binders – based on the premise that ‘sticky’ proteins are detected more often than proteins binding highly selectively to specific target proteins used as baits.

## Concluding remarks

MS-based proteomics technology, which is already of value for cell biology, has great scope for further expansion and is poised to deliver major new insights into biological responses and molecular regulatory mechanisms. Over the coming years we expect that the increasing availability of benchtop MS instruments and widening of the expertise base will allow proteomics to become a standard tool in many cell biology laboratories rather than remaining the exclusive domain of specialised facilities. Further improvements in the speed and resolution of MS instruments, combined with improved experimental design and new data analysis methods, should help to make proteomics widely available as an assay tool for quantitating a wide range of protein properties and responses, and not merely a means of protein identification. Furthermore, as well as comparing relative changes in protein levels under different conditions, MS-based methods are also being developed to allow absolute quantitation of protein levels; this will be extremely useful for building a detailed systems model of many cellular reactions and *in vivo* signalling pathways 39, 40, 41, 42.

Proteomics is fast approaching the ability to sample a ‘complete proteome’, whereby a ‘complete proteome’ can be defined as detection of peptide data for all expressed genes [43]. However, current proteomics technology still falls short of the capability to detect full, expressed protein sequences comprehensively. Thus, even the deepest coverage in the most detailed proteomics studies at present detects less than 50% of the peptides in expressed proteins. The ability to identify this ‘dark matter’ of the cell proteome promises to reveal exciting new information. For example, it may reveal peptides with complex PTMs that contribute to the interactions and regulation of the cognate proteins. Another interesting opportunity for new technical advances is in the area of ‘top down’ approaches to MS-based protein identification. Although most cell biology-related proteomics work to date has concentrated on the ‘bottom-up’ approach, where proteins are identified indirectly via detection of digested peptide fragments (Figure 1), it is now becoming feasible to resolve accurately by MS very large ions that correspond to intact protein molecules [44]. Future generations of mass spectrometers that extend this capability could thus offer exciting opportunities for direct protein analyses that could overcome some of the current limitations and ‘data averaging’ issues inherent in the bottom-up approach.

Maximising the future value to the cell biological community of the burgeoning proteomics data mountain requires new initiatives to promote the effective sharing and integration of data. We look forward to such developments, and suggest it would be most effective if led directly by cell biologists themselves and managed at the international community level with stable long-term funding. In another 15 years, therefore, it may well be that cell biology students will need to be as familiar with MS instrumentation and computers as they currently are with microscopes.
